# Haematological and plasma biochemistry reference intervals for free-ranging Australian pelicans

**DOI:** 10.1093/conphys/coag042

**Published:** 2026-08-03

**Authors:** D A Sullivan, J L Porter, J Ward, D Ramp, K J Brandis

**Affiliations:** Centre for Compassionate Conservation, Transdisciplinary School, University of Technology Sydney, 15 Broadway, Ultimo, New South Wales 2007, Australia; BirdLife Australia, Level 2, Main Building, 54 Wellington Street, Collingwood, Victoria 3066, Australia; Department of Climate Change, Energy the Environment & Water, 4 Parramatta Square, 12 Darcy Street, Parramatta, New South Wales 2150, Australia; Centre for Ecosystem Science, School of Biological, Earth and Environmental Sciences, University of New South Wales, High Street, Kensington, New South Wales 2052, Australia; Metung Veterinary Clinic, Hardys Road, Metung, Victoria 3904, Australia; Centre for Compassionate Conservation, Transdisciplinary School, University of Technology Sydney, 15 Broadway, Ultimo, New South Wales 2007, Australia; Centre for Ecosystem Science, School of Biological, Earth and Environmental Sciences, University of New South Wales, High Street, Kensington, New South Wales 2052, Australia

**Keywords:** Avian, biochemistry, Gippsland Lakes, haematology, packed cell volume, pelican, Ramsar, reference intervals

## Abstract

The aim of this study was to establish haematological and plasma biochemistry reference intervals for free-ranging adult and pre-fledged juvenile Australian pelicans (*Pelecanus conspicillatus*). These values represent the first species-specific assessments conducted to establish baseline blood values in a wild population of this species. Blood samples were collected from 58 adult pelicans and 53 pre-fledged juveniles between 2018 and 2024 across key sites in the Gippsland Lakes, Victoria, a significant Ramsar wetland for breeding pelicans. Complete blood counts and plasma biochemistry analyses were conducted to characterize reference intervals. White blood cell counts showed heterophils as the most abundant cell type in all age classes. Results revealed significant age-related differences in haematocrit values and plasma chemistry, including urea, total protein and calcium. By interpreting these data within a physiological as well as pathological framework, the study enhances understanding of normal species function and variation across life stages. This study fills a critical gap in understanding pelican physiology and provides essential reference data for wildlife veterinarians, rehabilitators and conservation biologists in the assessment and management of pelican health, facilitating more effective interventions and providing a tool for monitoring broader ecosystem health.

## Abbreviations

A/G: albumin/globulin ratio
ALP: alkaline phosphatase
AST: aspartate aminotransferase
CBC: complete blood count
CK: creatine kinase
Cl: chloride ions
GGT: gamma-glutamyl transferase
K: potassium
Na: sodium
PCR: polymerase chain reaction
PCV: packed cell volume
WBC: white blood cell

## Introduction

The Australian pelican (*Pelecanus conspicillatus*) is the only pelican species native to Australia. It is one of eight species of pelicans (Family—Pelecaniformes), with a range covering Australia, Papua New Guinea, Indonesia, Fiji and New Zealand. It is one of Australia’s most iconic and widespread waterbirds, occupying both coastal and inland wetlands where large-scale breeding is driven by unpredictable rainfall events (Kingsford & Norman, 2002). The Australian pelican is currently described as ‘least concern’ by the International Union for Conservation of Nature (IUCN); however, long-term data show significant declines across south-eastern Australia over the last 40 years (Kingsford *et al.,* 2020). These declines have been attributed to reduced opportunities and habitat for breeding due to climate change and river regulation (Gibson *et al.,* 2002; O'Brien *et al.,* 2010), as well as direct challenges faced by burgeoning human populations that compete with pelicans for food (i.e. fish), create disturbances through boating and other aquatic activities (Victorian Fisheries Authority, 2020) and pollute waterways (Khan *et al.,* 2023; Wells *et al.,* 2024) Interactions between pelicans and people can result in negative outcomes, requiring pelicans to receive assistance from conservation agencies, wildlife rescuers and carers, veterinarians and zoos (Carapetis *et al.,* 2014; Miller *et al.,* 2022). Pelicans experience injuries from entanglement in fishing tackle, from dog attacks, and can suffer poor health from inappropriate dietary items, including foraging in waste management facilities (municipal dumps) (Kohler, 2015; Forys *et al.,* 2026).

Individual pelicans exposed to these different challenges may exhibit both chronic and acute stress, expressed as complex reactions to both anthropogenic and environmental circumstances (Jodice *et al.,* 2022). Exposure to such stressors frequently results in measurable physiological changes that impact their health, behaviour, movement and reproductive ecology (Jodice *et al.,* 2022). Physiological changes, and their haematology and blood biochemistry markers, can therefore be used to infer changes in population health and ecosystem conditions, enhancing our understanding of the mechanisms underlying the status and trends of wildlife populations (Loss *et al.,* 2012; Maceda-Veiga *et al.,* 2015) and informing conservation strategies. Studies on other pelican species outside of Australia have explored reproductive success, health, impacts of human coexistence on populations and associated food resources (Potti *et al.,* 1999; Sachs & Jodice, 2009; Ferguson *et al.,* 2014). The relationship between species-specific blood chemistry and environmental influences is increasingly being investigated as a monitoring tool for avian health and environmental stressors (Cooke *et al.,* 2017; Madliger *et al.,* 2018; Ottinger *et al.,* 2019; Jodice *et al.,* 2022; Kim *et al.,* 2025).

Although Australian pelicans are increasingly presenting for care (Marine Response Unit - Melbourne Zoo, 2024 personal communication), there is a significant knowledge gap in how injuries and negative anthropogenic interactions alter pelican physiology, potentially impeding their recovery and restricting care options. Reference intervals (RIs) for haematology and biochemistry are often extrapolated from studies on other pelican species such as brown pelican (*Pelecanus occidentalis*) (Ferguson *et al.,* 2014; Jodice *et al.,* 2022) to guide clinical decision-making for Australian pelicans. However, geographical, physiological and behavioural traits between species are different enough to potentially alter blood chemistry.

This study aimed to establish haematological and plasma biochemistry RIs for free-ranging Australian pelicans. These values represent the first foundational blood parameters defined for a wild population of this species. As large waterbirds occupying high trophic levels, Australian pelicans reflect both individual and ecosystem-level pressures through their physiological responses. Accordingly, the RIs presented here provide essential baseline data to assist biologists, rehabilitators and veterinarians in clinical assessment and management, while also supporting long-term ecological monitoring and evaluation of population and ecosystem health.

## Materials and methods

### Study area

**Figure 1:**
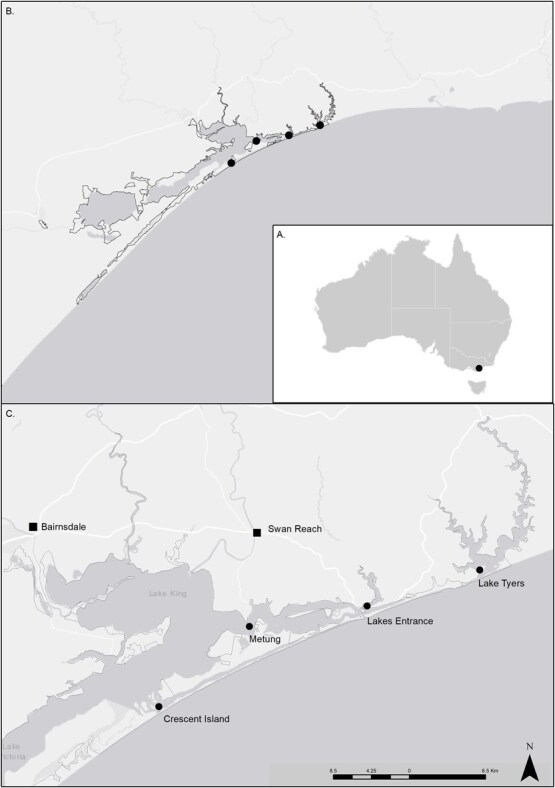
Study area for this research. (**a**) The Gippsland Lakes. Located in south-eastern Victoria, Australia. (**b**) The Ramsar boundary of the Gippsland Lakes, with capture locations indicated by dot points. (**c**) Capture sites are located in Metung, Lakes Entrance, Lake Tyers and Crescent Island (rookery). The locations of nearby townships are shown using square icons

The Gippsland Lakes, located in south-eastern Victoria (Fig. 1), are a Ramsar wetland of international significance (Kirono *et al.,* 2022; East Gippsland Catchment Management Authority, 2024) and are the largest navigable estuarine lagoon system in Australia, covering 61 150 hectares (Kirono *et al.,* 2022). The Gippsland Lakes are an internationally important coastal wetland system (Kirono *et al.,* 2022; East Gippsland Catchment Management Authority, 2024) under extreme pressure from a range of anthropogenic factors, including urban development, commercial fishing and recreational disturbance (Boon, 2014; Victorian Fisheries Authority, 2020; Kirono *et al.,* 2022). The Australian pelican is an important part of the area’s cultural and biodiversity values, playing a key role in this wetland ecosystem as a top-order predator.

The Gippsland Lakes contain one of two remaining permanent pelican rookeries in the State of Victoria from a historical ten (O'Brien *et al.,* 2010). Loss of critically important breeding sites elsewhere has been accompanied by a geographic (distributional) decline as recently as 2006, leaving the Gippsland Lakes as an increasingly important wetland for pelicans. The Lakes also hold conservation and environmental values for local communities and indigenous cultural significance.

### Capture

Preliminary fieldwork at the study site found that the capture of pre-fledged young Australian pelicans could be tolerated by the rookery without loss or abandonment of nests (Ferguson *et al.,* 2014), with adults returning to eggs and young rapidly after disturbance (pers. obs.). Consequently, pre-fledged juvenile pelicans (10–14 weeks old) were sampled from rookeries at Crescent Island (Fig. 1) during the breeding seasons 2018, 2022 and 2023. Individuals were gently herded into temporary holding pens where they were removed individually by hand and had their blood sampled (see below). Adult and sub-adult pelicans were captured by hand at three locations: Lake Tyers, Metung and Lakes Entrance (Fig. 1) between 2018 and 2024. Individuals were hand-captured throughout the year, with sampling conducted during all seasons (Supplementary F).

All birds were observed for abnormal behaviour (e.g. limping, uncoordinated movement, etc.) or for clinical signs of being unhealthy prior to their capture to ensure they were not selected for sampling. In addition, each bird was weighed and assigned a body condition score on a five-point scale (1 = emaciated, 2 = poor, 3 = fair, 4 = good, 5 = excellent) based on pectoral muscle mass and keel prominence, following standard avian condition scoring guidelines. Individuals scoring ≤3 (fair/poor/emaciated) were not included in RI calculations to ensure that the dataset represented ‘healthy’ individuals (Duerr *et al.,* 2013; Hanauska-Brown *et al.,* 2017). Individuals included in this study were therefore regarded as representative of a ‘healthy’ wild population. Due to the nature of capturing large free-ranging birds, capture opportunities were highly irregular and could not be conducted to ensure sampling occurred at the same time each day. Not all samples were suitable for complete analyses, and so sample sizes vary among blood analytes.

### Blood sampling

Blood samples were collected from either the lateral tarsal vein or medial metatarsal vein using 22-gauge, 1″ needles with a 3-ml syringe. We collected 1–2 ml of blood, well below the recommended body mass threshold of less than 1% (Sabater & Forbes, 2015). Extracted blood was collected via venipuncture in 5-ml lithium heparin tubes for transport to the laboratory. Samples were refrigerated at 4°C and analysed within 24–48 hours. Studies investigating the viability of blood cells from Black Storks (*Ciconia nigra*) following venipuncture for analysis show cell integrity was maintained within this timeframe (Puerta *et al.,* 1989). We recognize that whole-blood parameters can be affected by degenerative changes such as haemolysis, which may occur under field conditions or during sample storage prior to analysis. Samples with visible haemolysis graded as ≥2+ were therefore excluded from the dataset. The laboratory reported no artefacts or integrity issues among the retained samples. Venipuncture in our study was performed within 5 minutes of capture and restraint to minimise the effects of handling on haematology and biochemistry values caused by an increase in corticosterone levels (Davis, 2005; Kinney, 2018). The venipuncture collection methodologies described here are widely used and well documented in the literature (Puerta *et al.,* 1991; Zaias *et al.,* 2000; Kramer & Harris, 2010; Low, 2012). Birds were released after adequate clotting at the venipuncture site and were observed for normal behaviour.

### Sex

Sex of adults was inferred based on sexual dimorphism determined by morphometric measurements, including bill length, bill depth and overall body size, taken at the time of sampling and compared with morphological data collected from 100 adults (Sullivan *et al*., 2026). Adults whose sex could not be easily determined by measurements, along with pre-fledged juveniles and sub-adults, were sexed using polymerase chain reaction (PCR) analysis by isolating genomic DNA from blood samples that were collected on Whatman Flinders Technology Associates (FTA) Classic paper. PCR testing was performed by DNA Solutions, Wantirna, Victoria, Australia. Breeding and non-breeding status were assessed based on plumage characteristics and soft tissue colouration, with particular attention to the gular pouch and eye ring, which are known to exhibit brighter colouration during the breeding season (Vestjens, 1977). Observations of known activity at the breeding rookery, including nesting and chick rearing, were also considered to support classification. Sub-adults were similarly identified by plumage and soft tissue features, focusing on the colouration of the tarsus, tibia, gular pouch and eye ring. This is consistent with immature plumage characteristics observed prior to full adult maturation at 4 years of age.

### Sample processing

To ensure consistency in the analyses, the same laboratory and technicians were used throughout the study. This approach minimizes the likelihood of variability caused by differences in laboratory methods or technologies would influence the result, suggesting that any observed variation is more likely attributable to true species diversity (Maxwell & Robertson, 1998; Samour & Hart, 2020).

Whole blood samples for haematology were processed by ACE Laboratory Services (Bendigo, Victoria, Australia), a National Association of Testing Authorities (NATA)-accredited laboratory. A Roche KX-21N analyser (Roche Diagnostics, Basel, Switzerland) was used for haemoglobin determination only using direct current (DC) detection. Since automated analysers are not validated for avian leucocytes, all other haematological parameters were determined manually. Peripheral blood films were prepared from fresh samples, air-dried, fixed and stained using a Rapid Diff protocol consisting of fixative, eosin (red) and methylene blue (purple), then rinsed, dried and examined by an experienced laboratory scientist using an Olympus CH-2 microscope (Olympus Corporation, Tokyo, Japan). Haematocrit/packed cell volume (PCV) was determined by centrifugation of capillary tubes (850 rpm, 6 minutes), and blood films were prepared, fixed and stained for leucocyte evaluation. Total white blood cell (WBC) counts were estimated microscopically under ×40 magnification by averaging leucocytes across 10 monolayer fields and applying the standard formula. Differential counts were performed by classifying 100 leucocytes into heterophils, lymphocytes, monocytes, eosinophils or basophils, and percentages were applied to the estimated WBC to calculate absolute values.

### Biochemical analysis of plasma samples

After the haematological preparations were completed, plasma samples were obtained by centrifuging blood at 3000 rpm for 7 minutes. The plasma was then aliquoted and analysed using two different instruments. Electrolytes, including sodium (Na), potassium (K) and chloride (Cl), were measured using a Biobase BKE Analyser (BIOBASE Group, Jinan, Shandong, China) and ion-selective electrode (ISE) technology. All remaining biochemical analytes were measured using a Biobase BK-400 automatic analyser (BIOBASE Group, Jinan, Shandong, China). The following methodologies were applied for each analyte: urea (urease– Glutamate Dehydrogenase method (GLDH)), total protein (Biuret method), albumin (bromocresol green (BCG) dye-binding method), globulin (calculated as total protein–albumin), albumin/ globulin (A/G) ratio (albumin divided by globulin), calcium (Arsenazo III method), phosphate (colorimetric method), creatine kinase (CK) (International Federation of Clinical Chemistry and Laboratory Medicine method (IFCC)), aspartate aminotransferase (AST) and alkaline phosphatase (ALP) (IFCC methods), amylase (Ethylidene-Nitrophenyl-Maltoheptaoside method (EPS-G7)), uric acid (Tribromo-Hydroxybenzoic Acid method (TBHBA)) , total cholesterol and lipase (enzymatic colorimetric methods), bicarbonate (enzymatic method), gamma-glutamyl transferase (GGT) (Szasz method) and total bilirubin (vanadate oxidation method; excluded from interpretation due to lack of physiological relevance in birds) (Harr, 2002). All biochemical analyses were conducted on plasma samples following the completion of haematology assessments.

### Analytical considerations

While the BCG method used for albumin determination may overestimate avian albumin due to cross-reactivity with globulins, the A/G ratio was retained in this study as a physiologically informative parameter rather than a clinical diagnostic one (Harr, 2002; Whitehead, 2004; Sturkie, 2012; Williams, 2012).

### Statistical analysis

Descriptive analyses including mean, median, standard deviation (SD) and range were calculated for all haematological and biochemistry data across adult, sub-adult and juvenile age classes, as well as adult males and adult females (Supplementary A, B and C).

RIs for haematological and biochemical analytes were calculated in accordance with American Society for Veterinary Clinical Pathology (ASVCP) recommendations (Geffré *et al.,* 2011; Friedrichs *et al.,* 2012). Data were first examined visually using histograms to assess distribution and identify potential outliers. Formal outlier detection was conducted in RStudio and Reference Value Advisor© (RefVal), version 2.1 (National Veterinary School of Toulouse, Haute-Garonne, France). Outliers were excluded only when statistically and biologically justified; otherwise, all values were retained to preserve population variability.

Following outlier exclusion, RIs for each analyte were calculated using Reference Value Advisor Software. Data normality was assessed with the Anderson–Darling test, considering *P* > .05 as normally distributed. Ninety percent confidence intervals (CIs) were determined to define the upper and lower limits of the RIs, following ASVCP recommendations. For analytes with *n* ≥ 120, non-parametric methods were used. When 40 ≤ *n* < 120, either parametric or non-parametric approaches were applied depending on data distribution. For non-Gaussian data, bootstrap methods were used to estimate CIs. In cases where 20 ≤ *n* < 40, parametric or robust techniques were applied to either Box–Cox transformed or untransformed data. For sample sizes of 10 ≤ *n* < 20, only descriptive statistics (mean, SD, median, minimum and maximum) were generally reported. These procedures were conducted in accordance with guidelines from the International Federation for Clinical Chemistry and the Clinical and Laboratory Standards Institute (Horowitz, 2008; Horowitz, 2015). We used Kruskal–Wallis tests to assess potential differences in analyte values across sampling years before pooling the data for analysis. We conducted Wilcoxon rank-sum (Mann–Whitney U) tests (*P* < .05) to assess potential statistical differences in PCV values between adults and juveniles, between adult male and female birds and between breeding and non-breeding adults of both sexes. Wilcoxon rank-sum (Mann–Whitney U) tests were also used for comparison between sub-adult and juvenile PCV and sub-adult and adult PCV. Sub-adults were limited to PCV comparison only due to the low sample size.

Comparisons between blood analytes of adults and juveniles were performed using either independent samples *t*-tests, or Mann–Whitney U tests depending on data distribution. All analyses were two-tailed with a significance level of *P* < .05. *P* values between .05 and .10 were considered suggestive trends and interpreted in the context of the study’s exploratory nature. Analysis was conducted using DATAtab (DATAtab e.U., Graz, Austria) and RStudio (Posit Team, 2025).

### Ethics statement

This study is one component of a broader research project focusing on the movement, population, health and ecology of Australian pelicans within the Gippsland Lakes Ramsar site, Victoria, Australia. This study was conducted under Animal Ethics Committee permits from Phillip Island Nature Park #62018, #32021, #62024, Department of Energy, Environment and Climate Action Wildlife Research permit #10010090, #10011180 and Parks Victoria Access Agreements.

## Results

We collected blood samples for complete blood counts (CBCs) from 57 adults (33 females, 18 males and 6 birds of unknown sex) across both breeding and non-breeding seasons, six sub-adults (three males and three females) and 54 pre-fledged juveniles (1 female, 3 males and 51 unknown). Of the sub-adults (12–24 months; *n* = 6, both sexes), only three were included in PCV analysis, whereas all six were included in biochemical analyses. In some cases, no usable blood film was obtained, leaving only plasma for biochemical analysis, resulting in reduced CBC sample sizes. Although the sub-adult sample size was small (*n* = 3), the coefficient of variance of 9.18% supported inclusion of sub-adults in comparative PCV analysis. Other data for sub-adults (*n* = 6) were included to provide biological context and to explore potential developmental trends between juvenile and adult pelicans, rather than to establish definitive RIs.

Not all samples were suitable for complete analyses, so sample sizes vary among blood analytes. RIs for haematology and biochemistry values of Australian pelican adults appear in Table 1 and pre-fledged juveniles appear in Table 2. Values for sub-adults are in Supplementary A.

**Table 1:** Haematology and biochemistry RIs for adult free-ranging Australian pelicans sampled between 2018 and 2024 in Gippsland Lakes, Victoria

				**Adults**									
**Haematology (units)**	***n***	**Mean**	**SD**	**Median**	**Min**	**Max**	**And–Darl. Test**	**Dist.**	**Method**	**LRL of RI**	**URL of RI**	**CI 90% of LRL**	**CI 90% of URL**
PCV %	45	41	4	42	34	54	0.060	G	P	36	50	34–36	45–54
Haemoglobin g/L	44	166	14	167	140	206	0.869	G	P	143	193	140–147	186–206
Plasma protein g/L	43	41.1	10.4	42.0	1.8	68.0	0.000	NG	NP	13.0	53.9	1.8–32.1	50.0–68.0
WBC (estimated) × 109/L	40	10.5	4.8	9.3	3.4	26.0	0.070	G	P	3.8	20.1	3.4–5.6	16.7–26.0
Heterophils %	39	74	16	79	39	96	0.019	NG	NP	39	95	39–51	91–96
Lymphocytes %	40	24	17	18	2	67	0.002	NG	NP	3	60	2–8	47–67
Monocytes %	33	3	5	2	0	28	0.000	NG	NP	1	14	0–1	5–28
Eosinophils %	20	1	1	0	0	2	0.000	NG	NP	0	2	0–0	1–2
Basophils %	35	0	0	0	0	0	NA	NG	OR	0	0		
Biochemistry (units)													
Urea mmol/L	50	2.8	1.9	2.3	0.9	10.5	0.000	NG	NP	1.1	8.8	0.9–1.3	4.9–10.5
Total protein g/L	53	43.4	8.4	43.0	28.0	73.0	0.024	NG	NP	29.9	65.7	28.0–34.3	52.4–73.0
Albumin g/L	55	21.1	4.8	21.0	8.0	33.0	0.017	NG	NP	11.3	31.6	8.0–15.7	27.6–33.0
Globulin g/L	53	22.0	7.0	21.0	6.0	46.0	0.252	G	P	12.0	36.2	6.0–14.0	30.7–46.0
A/G ratio	53	1.08	0.55	0.90	0.40	3.80	0.000	NG	NP	0.50	2.44	0.40–0.69	1.60–3.80
Calcium mmol/L	52	2.6	0.7	2.4	1.9	6.0	0.000	NG	NP	1.9	4.0	1.9–2.1	3.4–6.0
Phosphate mmol/L	49	1.6	1.1	1.4	0.1	4.5	0.011	NG	NP	0.2	4.0	0.1–0.3	3.1–4.5
CK U/L	51	2208	1234	2087	7	9249	0.000	NG	NP	324	4702	7–1398	2642–9249
AST U/L	55	232	127	201	120	912	0.000	NG	NP	125	468	120–134	414–912
Amylase U/L	54	2543	568	2477	1522	3804	0.151	G	P	1606	3592	1522–1854	3446–3804
Uric acid mmol/L	40	970	543	920	145	2228	0.087	G	P	204	1871	145–318	1605–2228
Cholesterol mmol/L	47	4.8	1.1	4.9	1.1	7.6	0.403	G	P	3.2	6.9	1.1–3.7	6.0–7.6
Bicarbonate mmol/L	44	17.1	4.8	17.0	2.3	33.0	0.009	NG	NP	11.0	26.8	2.3–12.1	22.0–33.0
Lipase U/L	48	24	19	16	1	86	0.000	NG	NP	3	77	1–7	51–86
GGT U/L	48	8	11	6	1	72	0.000	NG	NP	1	33	1–1	18–72
ALP U/L	49	575	462	484	1	2533	0.000	NG	NP	19	1758	1–95	982–2533
Total bilirubin μmol/L	48	8.0	5.6	6.7	0.5	26.3	0.001	NG	NP	1.0	20.7	0.5–2.0	15.9–26.3
Sodium (Na) mmol/L	33	138.3	4.3	137.0	133.0	154.0	0.003	NG	NP	133.0	148.4	133.0–134.6	143.0–154.0
Potassium (K) mmol/L	33	9.3	1.9	8.8	5.3	14.7	0.279	G	P	5.8	13.0	5.3–7.6	11.1–14.7
Chloride (Cl) mmol/L	33	115.7	5.9	114.1	107.0	142.0	0.000	NG	NP	110.2	128.4	107.0–111.8	119.4–142.0

The number of samples analysed for each analyte varies due to either the sample volume available or outlier exclusion. Distribution: G = Gaussian; NG = non-Gaussian. Statistical method for establishing RI: P = parametric; NP = non-parametric; OR = observed range. Darl. Test = Anderson–Darling test; LRL = lower reference limit; URL = upper reference limit; CI = confidence interval. A/G ratio = albumin/globulin ratio; CK = creatine kinase; AST = aspartate aminotransferase; GGT = gamma-glutamyl transferase; ALP = alkaline phosphatase.

Eosinophils and basophils demonstrated a zero-inflated, non-Gaussian distribution. Given *n* = 20 and the instability of parametric and robust methods, the RI was defined using the observed range.

**Table 2:** Haematology and biochemistry RIs for pre-fledged juvenile free-ranging Australian pelicans sampled between 2018 and 2024 in Gippsland Lakes, Victoria

				**Juveniles**									
**Haematology (units)**	***n***	**Mean**	**SD**	**Median**	**Min**	**Max**	**And Darl. Test**	**Dist.**	**Method**	**LRL of RI**	**URL of RI**	**CI 90% of LRL**	**CI 90% of URL**
PCV %	54	31	3	32	25	39	0.026	NG	NP	26	38	25–27	35–39
Haemoglobin g/L	54	113	13	112	87	166	0.051	G	P	91	141	87–99	127–166
Plasma protein g/L	53	33.3	5.2	34.0	12.0	42.0	0.005	NG	NP	24.0	40.0	12.0–27.0	38.0–42.0
WBC (estimated) × 109/L	54	9.4	3.7	9.9	2.4	19.3	0.000	NG	NP	3.7	17.9	2.4–4.5	15.8–19.3
Heterophils %	53	66	16	69	20	93	0.000	NG	NP	23	84	20–39	83–93
Lymphocytes %	54	33	16	30	6	80	0.000	NG	NP	14	77	6–16	61–80
Monocytes %	21	2	2	2	1	9	0.000	NG	NP	1	7	1–1	4–9
Eosinophils %	8	2	3	1	0	8	0.003	NG	NP	0	7	0–0	1–8
Basophils %	37	0	0	0	0	0	NA	NG	OR	0	0		
Biochemistry (units)													
Urea mmol/L	41	2.2	2.8	1.5	0.8	15.1	0.000	NG	NP	0.8	12.1	0.8–0.9	2.6–15.1
Total protein g/L	45	37.5	5.5	37.0	30.0	61.0	0.003	NG	NP	30.1	46.0	30.0–32.1	44.8–61.0
Albumin g/L	46	22.9	2.9	23.0	12.0	29.0	0.003	NG	NP	18.1	28.0	12.0–20.1	26.9–29.0
Globulin g/L	45	14.4	3.8	14.0	10.0	32.0	0.000	NG	NP	10.0	22.8	10.0–11.0	17.0–32.0
A/G ratio	45	1.68	0.32	1.70	0.90	2.60	0.126	G	P	1.01	2.29	0.90–1.40	2.00–2.60
Calcium mmol/L	46	2.7	0.3	2.8	1.9	3.1	0.000	NG	NP	1.9	3.0	1.9–2.2	3.0–3.1
Phosphate mmol/L	46	2.7	0.6	2.7	0.2	4.2	0.016	NG	NP	1.9	3.6	0.2–2.1	3.4–4.2
CK U/L	45	3417	1119	3302	1728	9249	0.000	NG	NP	2188	4548	1728–2403	4123–9249
AST U/L	46	177	118	151	110	912	0.000	NG	NP	112	300	110–117	218–912
Amylase U/L	45	2150	332	2101	1265	2760	0.893	G	P	1487	2695	1265–1773	2628–2760
Uric acid mmol/L	43	493	310	439	118	1682	0.000	NG	NP	127	1337	118–199	805–1682
Cholesterol mmol/L	46	5.1	1.1	4.8	1.1	8.1	0.034	NG	NP	3.4	6.9	1.1–4.0	6.3–8.1
Bicarbonate mmol/L	46	17.3	3.1	17.5	10.0	23.0	0.340	G	P	10.2	22.0	10.0–13.0	21.0–23.0
Lipase U/L	44	50	38	56	8	161	0.000	NG	NP	8	104	8–9	95–161
GGT U/L	42	7	5	6	1	26	0.000	NG	NP	1	18	1–2	13–26
ALP U/L	42	556	211	562	68	965	0.695	G	P	190	940	68–315	821–965
Total bilirubin μmol/L	42	21.6	10.1	21.8	3.0	42.2	0.881	G	P	3.9	41.3	3.0–8.8	33.0–42.2
Sodium (Na) mmol/L	39	139.1	2.6	139.0	134.0	144.0	0.102	G	P	134.0	144.0	134.0–136.0	143.0–144.0
Potassium (K) mmol/L	39	5.7	1.3	5.5	4.1	11.9	0.000	NG	NP	4.2	8.4	4.1–4.5	6.3–11.9
Chloride (Cl) mmol/L	38	108.9	3.6	108.0	101.0	118.0	0.237	G	P	102.8	115.2	101.0–104.9	113.2–118.0

The number of samples analysed for each analyte varies due to either the sample volume available or outlier exclusion.

Distribution: G = Gaussian, NG = non-Gaussian. Statistical method for establishing RI: P, parametric; NP, non-parametric; OR = observed range. And. Darl. Test = Anderson–Darling test; LRL = lower reference limit; URL = upper reference limit; CI = confidence interval. A/G ratio = albumin/globulin ratio; CK = creatine kinase; AST = aspartate aminotransferase; GGT = gamma-glutamyl transferase; ALP = alkaline phosphatase

### Packed cell volume

**Figure 2:**
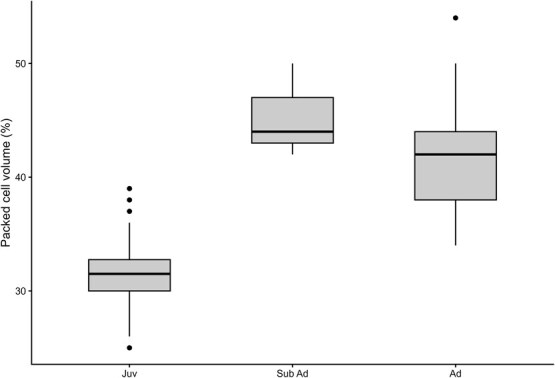
PCV % of blood samples from free-ranging Australian pelicans. Both sexes are represented in each age class

There was a statistically significant difference in PCV between adult (*n* = 45) and juvenile (*n* = 54) age groups (*W* = 2393.5, *P* < .001). Adult PCV was 32% higher than that of juveniles. Similarly, the PCV between sub-adults and juveniles was also statistically different (*W* = 0, *t*, *P* = .004), with sub-adults 45% greater than juveniles. The PCV of sub-adults and adults did not differ significantly (*W* = 106, *P* = .10) (Fig. 2).

The PCV analysis revealed no significant sex-based differences in adult birds (*W* = 154, *P* = .267), allowing us to pool the data for further analysis. Similarly, no statistical difference was observed in PCV between the sexes of juvenile birds (*W* = 0, *P* = .50), so these data were also pooled for further analysis. No interannual differences were observed among juveniles (Kruskal–Wallis test, *χ*^2^ = 5.20, df = 2, *P* = .074) sampled in 2018, 2023 and 2024; consequently, data from these years were pooled for analysis. No significant differences in PCV were detected between breeding and non-breeding adult females (breeding *n* = 18, non-breeding *n* = 8, *W* = 66, *P* = .76) or between breeding and non-breeding adult males (breeding *n* = 9, non-breeding *n* = 6; *W* = 37, *P* = .26).

### White blood cell count

WBC differential counts identified heterophils as the most abundant cells in both adult (74.3 ± 15.6%, *n* = 40) and juvenile pelicans (66.2 ± 15.9%, *n* = 53), followed by lymphocytes (adults: 24.2 ± 16.8%, *n* = 40; juveniles: 32.5 ± 16.2%, *n* = 54). Total WBC did not show a statistically significant difference between adults (10.5 ± 4.8 × 10^9^/L, *n* = 40) and juveniles (9.4 ± 3.7 × 10^9^/L, *n* = 54; *W* = 1196, *P* = .373). Descriptive statistics and RIs for adult and juvenile WBC parameters are presented in Tables 1 and 2, respectively.

However, significant differences were observed in leukocyte differentials: adults showed higher heterophil percentages (*W* = 1376, *P* = .007), and juveniles showed higher lymphocyte percentages (*W* = 706, *P* = .004). No significant age-related differences were detected between monocyte (*P* = .459) and eosinophil percentages (*P* = .322).

### Plasma biochemical values

Significant age-related differences occurred in several plasma biochemical analytes between adults and juveniles. Adults exhibited significantly higher levels of plasma protein, total protein, globulin, urea, AST, amylase, uric acid, potassium and chloride. In contrast, juveniles showed significantly higher A/G ratios, phosphate (juveniles: 2.68 mmol/L; adults: 1.63 mmol/L; *W* = 501.5, *P* < .001), CK (juveniles: 3284.7 U/L; adults: 2089.7 U/L; *W* = 136, *P* < .001), lipase, calcium and albumin concentrations. No significant age-related differences were detected for cholesterol, bicarbonate, GGT, ALP or monocyte and eosinophil percentages. Comprehensive statistical comparisons between adult and juvenile haematological and plasma biochemical analytes are presented in Supplementary E.

## Discussion

This study provides the first species-specific baseline of haematological and plasma biochemistry RIs for free-ranging Australian pelicans. These results provide a key primary resource for clinical veterinarians to interpret blood values for this species and allow for specific clinical pathological diagnosis. Together, these RIs provide a critical framework for clinicians and conservation practitioners, supporting species-specific interpretation of haematological and biochemical values and strengthening diagnostic and health-assessment capacity across clinical and ecological contexts.

No prior published research has examined the haematology and biochemistry of Australian pelicans. Consequently, existing RIs for clinical comparison have predominantly been extrapolated from confamilial species, including the brown pelican (*P. occidentalis*) (Zaias *et al.,* 2000; Maceda-Veiga *et al.,* 2015; Jodice *et al.,* 2022) and the great white pelican (*P. onocrotalus*) (Harr, 2002; Low, 2012; Sturkie, 2012). Many of these studies describe RIs drawn from captive or incapacitated individuals rather than wild populations; however, research is continuing to accumulate in this area, and sample sizes from wild populations continue to increase.

PCV is an essential diagnostic tool in avian medicine, offering valuable information on physiological imbalances, such as inflammatory responses, dehydration or altered nutritional condition (Jones, 2015) and supporting timely clinical decision-making (Clark *et al.,* 2009; Thrall *et al.,* 2012). Our data reveal that the mean PCV of Australian pelicans falls towards the lower end of reference ranges than that reported for other pelican species, although it still falls within the established normal intervals for those species (Supplementary D).

We identified lower PCV values in juveniles compared to adults, consistent with findings in studies on other pelican species (Wolf *et al.,* 1985; Vaz *et al.,* 2015; Jodice *et al.,* 2022). PCV levels are known to vary across different developmental stages, including nestlings, fledglings and sub-adults (Work, 1996; Fair *et al.,* 2007), likely attributable to the increased oxygen demands associated with development prior to fledging. We also observed elevated PCV levels in sub-adults, which may be attributed to haematopoiesis, where maturing individuals have not yet reached the stable red blood cell production seen in fully mature adults (Work, 1996; Shmueli *et al.,* 2000). Dispersal of sub-adult birds from the Gippsland Lakes area post-fledging contributed to the small sample size of this age class in the study. PCV values during the breeding season in both males and females are consistent with findings from other avian studies. Fair *et al.* (2007) also suggested that seasonal variation may influence PCV values in temperate climates due to increased energetic demands associated with thermoregulation and reproductive activity. Although the analysis of seasonal variation was beyond the scope of this study, the Gippsland Lakes region is classified as a temperate climate with significant seasonal differences (Bureau of Meteorology *et al.,* 2019; Ruuskanen *et al.,* 2021). Future studies investigating seasonal environmental conditions and breeding activity may provide further insight into physiological drivers of PCV variation in Australian pelicans.

Mean values of ALP in juveniles compared to adults were not statistically significant; however, they exhibited significantly higher CK and phosphate levels compared to adults. Research has shown that in birds, CK, ALP and phosphate levels are associated with rapid musculoskeletal development, osteoblastic activity and skeletal growth (Wolf *et al.,* 1985; Zaias *et al.,* 2000; Garcia *et al.,* 2010). Mean values of AST and GGT in juveniles did not exceed adult values, suggesting that increased phosphate may therefore be related to bone development in pre-fledged juveniles rather than hepatic enzyme induction (Wolf *et al.,* 1985; Harr, 2002; Garcia *et al.,* 2010). Research in brown pelicans suggests that bone formation may continue for up to 24 months, resulting in moderately elevated ALP values until skeletal maturity (Wolf *et al.,* 1985; Zaias *et al.,* 2000; Harr, 2002). In the present study, this is supported by a concurrent increase in PCV among sub-adults up to 24 months of age, reflecting continued red blood cell development and metabolic maturation consistent with growth and elevated osteoblastic activity rather than pathology (Harr, 2002; Garcia *et al.,* 2010; Sturkie, 2012; Williams, 2012). No difference in AST activity was detected between breeding and non-breeding adult females (*W =* 101, *P* = .88). Previously published research has reported that free-ranging adult brown pelicans had lower levels of AST, ALT and albumin (Jodice *et al.,* 2022) than captive birds, which may also reflect reproductive or nutritional influences rather than hepatic pathology. Reference values established in this study fill a crucial knowledge gap in the literature and enhance clinical diagnosis and wildlife management, aiding biologists, veterinarians and rehabilitators in the assessment and treatment of injured Australian pelicans. Equally, these data provide insight into normal physiological processes, offering a foundation for interpreting natural variation associated with growth, reproduction and environmental adaptation. By establishing species-specific RIs, this research supports both diagnostic application and the broader understanding of pelican physiology. Given the species’ long lifespan, high trophic position and reliance on aquatic food webs, Australian Pelicans are well suited as indicators of wetland ecosystem condition. Accordingly, this work provides a foundational resource for future health surveillance, ecological monitoring and conservation programs for this species.

## Supplementary Material

Web_Material_coag042

## Data Availability

Data available on request.
