# Supplementary material for: Haematological and plasma biochemistry reference intervals for free-ranging Australian pelicans
Source: Conserv Physiol. 2026 Aug 3;14(1):coag042. doi: 10.1093/conphys/coag042 (PMC13432993; doi:10.1093/conphys/coag042)
Supplement: Web_Material_coag042 [file web_material_coag042.pdf]

Supplementary:

Supplementary A: Haematology and biochemistry reference intervals for sub – adult, free-ranging Australian pelicans sampled between 2018-2024 in Gippsland Lakes, Victoria, Australia.

Reference intervals could not be established for some analytes due to insufficient sample size and failure to meet assumptions of normality and symmetry required for parametric and robust methods. In addition, sample sizes do not meet the ASVCP minimum requirements (n ≥ 40 for robust methods; n ≥ 120 for non-parametric methods), making non-parametric methods unsuitable. Several calculated intervals yielded biologically implausible negative limits. Therefore, no reference interval is reported; instead, summary statistics (median, minimum, maximum) are provided for descriptive purposes.

And. Darl. Test - Anderson–Darling test; LRL = lower reference limit; URL = upper reference limit; CI = confidence interval. A/G ratio = albumin/globulin ratio; CK = creatine kinase; AST = aspartate aminotransferase; GGT = gamma-glutamyl transferase; ALP = alkaline phosphatase.

		Sub-adults											
**Haematology (units)**	**n**	**Mean**	**SD**	**Median**	**Min**	**Max**	**And. – Darl. Test**	**Dist.**	**Method**	**LRL of RI**	**URL of RI**	**CI 90% of LRL**	**CI 90% of URL**
PCV %	3	45	4	44	42	50	NA	NG	OR	42	50	-	-
Haemoglobin g/L	3	176	11	180	163	184	NA	NG	OR	163	184	-	-
Plasma Protein g/L	2	52.0	14.1	52.0	42.0	62.0	NA	NG	OR	42.0	62.0	-	-
WBC (Estimated) x 109/L	3	11.6	9.0	6.8	6.0	22.0	NA	NG	OR	6.0	22.0	-	-
Heterophils %	3	71	18	81	51	82	NA	NG	OR	51	82	-	-
Lymphocytes %	3	26	18	17	14	47	NA	NG	OR	14	47	-	-
monocytes %	3	3	2	2	1	5	NA	NG	OR	1	5	-	-
Eosinophils %	1	0	NA	0	0	0	NA	NG	OR	0	0	-	-
Basophils %	3	0	0	0	0	0	NA	NG	OR	0	0	-	-
**Biochemistry (units)**													
Urea mmol/L	5	3.3	3.3	1.7	0.3	8.1	NA	NG	OR	0.3	8.1	-	-
Total Protein g/L	6	45.8	17.0	48.0	15.0	63.0	NA	NG	OR	15.0	63.0	-	-
Albumin g/L	6	22.5	8.7	23.0	8.0	34.0	NA	NG	OR	8.0	34.0	-	-
Globulin g/L	6	23.2	10.4	23.0	7.0	38.0	NA	NG	OR	7.0	38.0	-	-
A/G ratio	6	1.03	0.28	1.10	0.50	1.30	NA	NG	OR	0.50	1.30	-	-

Calcium mmol/L	6	2.4	0.3	2.4	2.0	2.9	NA	NG	OR	2.0	2.9	-	-
Phosphate mmol/L	5	1.9	1.0	1.5	1.0	3.6	NA	NG	OR	1.0	3.6	-	-
CK U/L	6	1534	1171	1968	9	2616	NA	NG	OR	9	2616	-	-
AST U/L	6	216	46	208	167	299	NA	NG	OR	167	299	-	-
Amylase U/L	5	2459	833	2617	1067	3292	NA	NG	OR	1067	3292	-	-
Uric Acid mmol/L	3	1054	426	1005	655	1503	NA	NG	OR	655	1503	-	-
Cholesterol mmol/L	5	4.0	0.9	4.1	2.5	4.7	NA	NG	OR	2.5	4.7	-	-
Bicarbonate mmol/L	5	19.8	4.5	18.0	16.0	26.0	NA	NG	OR	16.0	26.0	-	-
Lipase U/L	6	16	12	12	7	39	NA	NG	OR	7	39	-	-
GGT U/L	6	9	8	6	1	23	NA	NG	OR	1	23	-	-
ALP U/L	6	230	153	212	12	408	NA	NG	OR	12	408	-	-
Total Bilirubin umol/L	6	4.8	6.0	3.3	0.3	16.6	NA	NG	OR	0.3	16.6	-	-
Sodium (Na) mmol/L	4	141.4	5.3	141.0	136.0	147.5	NA	NG	OR	136.0	147.5	-	-
Potassium (K) mmol/L	4	6.9	1.8	7.0	4.8	8.7	NA	NG	OR	4.8	8.7	-	-
Chloride (Cl) mmol/L	4	113.6	2.9	113.0	111.0	117.5	NA	NG	OR	111.0	117.5	-	-

Supplementary B –Haematology and biochemistry reference intervals for adult male, free - ranging Australian pelicans sampled between 2018 - 2024 in Gippsland Lakes, Victoria.

Number of samples analysed for each analyte varies due to either the sample volume available or outlier exclusion. Distribution. G = Gaussian, NG = non-Gaussian. Statistical method for establishing RI: P, parametric; NP, non-parametric; OR = observed range. And. Darl. Test - Anderson–Darling test; LRL = lower reference limit; URL = upper reference limit; CI = confidence interval. A/G ratio = albumin/globulin ratio; CK = creatine kinase; AST = aspartate aminotransferase; GGT = gamma-glutamyl transferase; ALP = alkaline phosphatase.

Reference intervals for monocytes, eosinophils, and basophils could not be established due to insufficient sample size and failure of normality and symmetry assumptions required for parametric and robust methods. Non-parametric methods were not applicable.

**ND: Confidence interval for this limit could not be determined due to sample size and data distribution. These intervals should be considered provisional.*

Australian Pelican Adult Males

Haematology (units)	n	Mean	SD	Median	Min	Max	And. – Darl. Test	Dist.	Method	LRL of RI	URL of RI	CI 90% of LRL	CI 90% of URL
PCV %	15	41	3	42	36	46	0.613	G	P	36	46	36 - 39	44 - 46
Haemoglobin g/L	13	164	14	164	140	188	0.905	G	P	141	185	140 - 153	176 - 188
Plasma Protein g/L	15	39.8	9.4	42.0	12.0	50.0	0.016	NG	NP	19.0	50.0	12.0 - 35.4	46.5 - 50.0
WBC (Estimated) x 109/L	14	10.7	6.1	8.6	3.8	26.0	0.026	NG	NP	4.2	24.0	3.8 - 6.5	13.6 - 26.0
Heterophils %	14	75	17	78	39	96	0.371	G	P	43	95	39 - 63	91 - 96
Lymphocytes %	14	21	17	18	2	60	0.120	G	P	3	55	2 - 7	35 - 60
Monocytes %	11	4	3	3	1	10	0.125	G	P	1	9	1 - 1	5 - 10
Eosinophils %	7	1	1	1	0	2	NA	NG	OR	0	2	-	-
Basophils %	11	0	0	0	0	0	NA	NG	OR	0	0	-	-
**Biochemistry (units)**													
Urea mmol/L	18	3.0	1.9	2.5	1.1	9.6	0.001	NG	NP	1.3	7.4	1.1 - 1.7	4.0 - 9.6
Total Protein g/L	16	40.9	6.6	41.5	28.0	51.0	0.609	G	P	29.5	50.6	28.0 - 35.0	46.6 - 51.0
Albumin g/L	18	18.8	6.1	19.0	8.0	33.0	0.282	G	P	9.3	31.3	8.0 - 13.0	22.1 - 33.0
Globulin g/L	16	21.5	5.5	21.0	12.0	32.0	0.870	G	P	13.1	31.6	12.0 - 17.0	26.2 - 32.0
A/G ratio	16	1.00	0.56	0.90	0.40	2.80	0.005	NG	NP	0.44	2.28	0.40 - 0.62	1.23 - 2.80
Calcium mmol/L	17	2.5	0.5	2.4	1.9	4.1	0.001	NG	NP	1.9	3.6	1.9 - 2.2	2.6 - 4.1
Phosphate mmol/L	14	2.0	1.0	2.0	0.5	4.5	0.180	G	P	0.7	3.9	0.5 - 1.2	2.5 - 4.5
CK U/L	17	2559	1763	2097	1285	9249	0.000	NG	NP	1393	6607	1285 - 1920	2569 - 9249
AST U/L	18	232	178	182	120	912	0.000	NG	NP	122	646	120 - 139	281 - 912
Amylase U/L	17	2388	632	2347	1522	3804	0.378	G	P	1544	3698	1522 - 1861	2815 - 3804
Uric Acid mmol/L	14	956	554	846	304	2228	0.323	G	P	330	2016	304 - 477	1380 - 2228
Cholesterol mmol/L	15	4.3	1.2	4.4	1.1	5.9	0.203	G	P	1.8	5.8	1.1 - 3.5	5.3 - 5.9
Bicarbonate mmol/L	12	14.8	5.4	15.0	2.3	25.0	0.142	G	P	5.0	23.6	2.3 - 12.5	18.2 - 25.0
Lipase U/L	13	20	12	16	5	44	0.048	NG	NP	6	42	5 - 14	29 - 44
GGT U/L	13	5	5	4	1	18	0.028	NG	NP	1	15	1 - 2	8 - 18
ALP U/L	14	777	715	575	68	2533	0.013	NG	NP	95	2297	68 - 231	1351 - 2533
Total Bilirubin umol/L	14	5.7	4.2	5.1	1.0	15.5	0.011	NG	NP	1.3	14.9	1.0 - 2.4	6.9 - 15.5
Sodium (Na) mmol/L	10	139.6	3.7	140.0	135.0	147.0	0.432	G	P	135.2	146.1	135.0 - 136.2	141.6 - 147.0
Potassium (K) mmol/L	10	9.5	1.1	9.6	8.0	11.3	0.794	G	P	8.1	11.2	8.0 - 8.6	10.3 - 11.3
Chloride (Cl) mmol/L	10	116.8	4.6	115.0	113.0	125.0	0.005	NG	NP	113.0	125.0	113.0 - 114.0	117.6 - 125.0

Supplementary C – Haematology and biochemistry reference intervals for adult female, free - ranging Australian pelicans sampled between 2018 - 2024 in Gippsland Lakes, Victoria.

Number of samples analysed for each analyte varies due to either sample volume available or outlier exclusion. Distribution: G = Gaussian, NG = non-Gaussian. Statistical method for establishing RI: P, parametric; NP, non-parametric; OR = observed range. And. Darl. Test = Anderson–Darling test; LRL = lower reference limit; URL = upper reference limit; CI = confidence interval. A/G ratio = albumin/globulin ratio; CK = creatine kinase; AST = aspartate aminotransferase; GGT = gamma-glutamyl transferase; ALP = alkaline phosphatase.

Reference intervals for monocytes, eosinophils, and basophils could not be established due to insufficient sample size and failure of normality and symmetry assumptions required for parametric and robust methods. Non-parametric methods were not applicable.

**ND: Confidence interval for this limit could not be determined due to sample size and data distribution. These intervals should be considered provisional.*

				Australian Pelican Adult Females									
**Haematology (units)**	**n**	**Mean**	**Median**	**SD**	**Min**	**Max**	**And. – Darl. Test**	**Dist.**	**Method**	**LRL of RI**	**URL of RI**	**CI 90% of LRL**	**CI 90% of URL**
PCV %	26	40	3	40	34	44	0.103	G	P	35	44	34 - 37	44 - 44
Haemoglobin g/L	27	164	11	165	145	186	0.477	G	P	146	183	145 - 150	176 - 186
Plasma Protein g/L	25	40.2	10.0	40.0	1.8	54.0	0.001	NG	NP	19.9	52.8	1.8 - 34.4	50.0 - 54.0
WBC (Estimated) x 109/L	22	10.6	4.2	9.8	3.4	19.3	0.891	G	P	4.1	18.1	3.4 - 6.2	15.6 - 19.3
Heterophils %	21	72	16	78	39	90	0.047	NG	NP	43	90	39 - 52	88 - 90
Lymphocytes %	22	28	17	22	8	67	0.043	NG	NP	9	60	8 - 11	49 - 67
Monocytes %	19	3	6	2	0	28	0.000	NG	NP	0	18	0 - 1	4 - 28
Eosinophils %	12	1	1	0	0	2	0.004	NG	NP	0	2	0 - 0	1 - 2
Basophils %	20	0	0	0	0	0	NA	NG	OR	0	0	-	-
**Biochemistry (units)**													
Urea mmol/L	26	2.6	2.0	1.9	1.1	10.5	0.000	NG	NP	1.2	7.0	1.1 - 1.4	3.8 - 10.5
Total Protein g/L	31	43.6	8.1	43.0	29.0	69.0	0.255	G	P	31.2	60.8	29.0 - 35.5	52.8 - 69.0
Albumin g/L	31	22.0	3.5	22.0	17.0	32.0	0.028	NG	NP	17.0	31.2	17.0 - 18.0	25.5 - 32.0
Globulin g/L	31	21.6	6.9	22.0	6.0	38.0	0.723	G	P	10.5	32.8	6.0 - 13.5	28.5 - 38.0
A/G ratio	31	1.13	0.59	0.90	0.70	3.80	0.000	NG	NP	0.70	2.15	0.70 - 0.70	1.60 - 3.80
Calcium mmol/L	30	2.5	0.5	2.3	2.0	3.9	0.000	NG	NP	2.1	3.6	2.0 - 2.2	3.2 - 3.9
Phosphate mmol/L	30	1.4	1.1	1.2	0.1	3.4	0.002	NG	NP	0.2	3.3	0.1 - 0.3	3.0 - 3.4
CK U/L	29	1892	630	2087	7	2686	0.000	NG	NP	19	2648	7 - 1367	2405 - 2686
AST U/L	31	223	84	212	126	469	0.001	NG	NP	126	446	126 - 139	299 - 469
Amylase U/L	31	2661	557	2601	1669	3617	0.401	G	P	1714	3546	1669 - 2030	3418 - 3617
Uric Acid mmol/L	21	988	572	904	145	1862	0.069	G	P	212	1828	145 - 342	1634 - 1862
Cholesterol mmol/L	27	5.1	0.9	5.2	3.4	6.9	0.840	G	P	3.6	6.7	3.4 - 4.0	6.0 - 6.9
Bicarbonate mmol/L	27	16.9	3.2	17.0	11.0	25.0	0.519	G	P	11.0	23.0	11.0 - 13.0	20.0 - 25.0
Lipase U/L	29	22	19	14	1	81	0.000	NG	NP	2	66	1 - 7	46 - 81

GGT U/L	29	10	14	8	1	72	0.000	NG	NP	1	45	1 - 2	18 - 72
ALP U/L	29	522	281	484	1	1292	0.466	G	P	41	1089	1 - 221	786 - 1292
Total Bilirubin umol/L	28	9.4	6.1	8.4	0.5	26.3	0.181	G	P	0.9	22.7	0.5 - 3.3	16.0 - 26.3
Sodium (Na) mmol/L	19	138.4	4.5	137.0	133.0	154.0	0.001	NG	NP	133.4	149.1	133.0 - 135.4	141.0 - 154.0
Potassium (K) mmol/L	19	8.9	1.9	8.7	5.3	12.6	0.887	G	P	5.6	12.4	5.3 - 7.0	10.4 - 12.6
Chloride (Cl) mmol/L	19	116.3	6.5	115.0	111.0	142.0	0.000	NG	NP	111.9	131.2	111.0 - 113.0	117.5 - 142.0

Supplementary D - PCV (%) of Pelican species found in published literature. Italics denote this study. * *Median reported instead of mean.*

Species	n	Mean	Range	Age	Status	Author/s (Year)
American White Pelican *(Pelecanus erythrorhynchos)*		43.6	31.4 - 56.0	N/A	N/A	Fowler's, Ch 12, Vol 8 (31)
Australian Pelican (Pelecanus conspicillatus)	*47*	*41*	*34 - 50*	*Adult*	*wild*	*This study*
Australian Pelican	*3*	*45*	*42 - 50*	*Sub-adult*	*wild*	*This study*
Australian Pelican	*53*	*31*	*25 - 50*	*Juvenile*	*wild*	*This study*
Brown Pelican *(Pelecanus occidentalis)*	70	45.8	38.5 - 52.9	Adult	wild	Jodice et al., 2022 (3)
Brown Pelican	5	46	43 - 49	Adult	captive	Samour, 2020 (20)
Brown Pelican	53	30	22 - 48	Juvenile	wild	Ferguson et al., 2014 (6)
Brown Pelican		45.7	33 - 59.9	N/A	N/A	Fowlers, Ch 12, Vol 8 (31)
Brown Pelican	66	46.6	33 - 54	Juvenile	captive	Gessner-Knepel, 2024 (Gessner-Knepel *et al.*, 2024)
Brown Pelican	2	41.9	N/A	Juvenile	captive	Wolf et al., 1985 (26)
Brown Pelican	6	45.0	N/A	Adult	captive	Wolf et al., 1985 (26)

Brown Pelican	5	43.1	N/A	Sub-adult	captive	Wolf et al., 1985 (26)
Great White Pelican *(Pelecanus onocrotalus)*	5	39.1	N/A	Adult	wild	Shmueli, 2000 (22)
Great White Pelican	6	41.5	N/A	Adult	captive	Puerta et al., 1991
Peruvian Pelican *(Pelecanus thagus)*	35	45.0*	40 - 49	Adult	wild	Watson et al., 2023 (Watson *et al.*, 2021)
Peruvian Pelican	9	34*	7-38	Juvenile	wild	Watson et al., 2023 (Watson, Langan, Allender, Cardeña, Cárdenas-Alayza and Adkesson, 2021)
Pink-backed Pelican *(Pelecanus rufescens)*		43	28.9 - 56	N/A	N/A	Fowlers, Ch 12, Vol 8 (31)

Supplementary E – Analyte comparisons between adults and juveniles using Wilcoxon rank-sum tests. $P < 0.05$ was considered statistically significant; P values between 0.05 and 0.10 were interpreted as marginal trends.

**Variable**	**Adult_n**	**Juvenile_n**	**Adult_mean**	**Juvenile_mean**	**W**	**P_value**	**Significance**
PCV %	45	54	41	31	2393.5	<0.001	Significant
Haemoglobin g/L	44	54	166	113	2353.5	<0.001	Significant
Plasma Protein g/L	43	53	41.1	33.3	1920.5	<0.001	Significant
WBC (Estimated) x 10^9/L	40	54	10.5	9.4	1196.5	0.373	Not significant
Heterophils %	39	53	74	66	1376.0	0.007	Significant
Lymphocytes %	40	54	24	33	705.5	0.004	Significant
Monocytes %	33	21	3	2	387.5	0.459	Not significant
Eosinophils %	20	8	1	2	61.5	0.322	Not significant
Basophils %	35	37	0	0	647.5	NaN	Not significant
Urea mmol/L	50	41	2.8	2.2	1578.0	<0.001	Significant
Total Protein g/L	53	45	43.4	37.5	1784.0	<0.001	Significant
Albumin g/L	55	46	21.1	22.9	853.5	0.005	Significant
Globulin g/L	53	45	22.0	14.4	2057.5	<0.001	Significant
A/G ratio	53	45	1.08	1.68	254.5	<0.001	Significant

Calcium mmol/L	52	46	2.55	2.67	711.5	<0.001	Significant
Phosphate mmol/L	49	46	1.63	2.68	501.5	<0.001	Significant
CK U/L	51	45	2208	3417	224.5	<0.001	Significant
AST U/L	55	46	232	177	1835.0	<0.001	Significant
Amylase U/L	54	45	2543	2150	1720.5	<0.001	Significant
Uric Acid mmol/L	40	43	970	493	1317.5	<0.001	Significant
Cholesterol mmol/L	47	46	4.8	5.1	935.5	0.265	Not significant
Bicarbonate mmol/L	44	46	17.1	17.3	918.0	0.449	Not significant
Lipase U/L	48	44	24	50	694.0	0.005	Significant
GGT U/L	48	42	8	7	1006.5	0.994	Not significant
ALP U/L	49	42	575	556	923.5	0.403	Not significant
Total Bilirubin umol/L	48	42	8.0	21.6	233.0	<0.001	Significant
Sodium (Na) mmol/L	33	39	138.3	139.1	463.0	0.041	Significant
Potassium (K) mmol/L	33	39	9.3	5.7	1217.0	<0.001	Significant
Chloride (Cl) mmol/L	33	38	115.7	108.9	1121.5	<0.001	Significant

Supplementary F – Year, age class and catch rates. Juvenile samples include creche-aged birds sampled from the breeding colony. Adult and sub-adult birds were sampled opportunistically at four capture sites across the Gippsland Lakes region

Year	Juvenile	Adult	Sub Adult	Total
2018	9	0	0	9
2019	0	7	1	8
2020	0	8	1	9
2021	0	2	0	2
2022	30	8	1	39
2023	16	29	2	47
2024	0	3	1	4
